# Pharmacogenetic effects of angiotensin-converting enzyme inhibitors over age-related urea and creatinine variations in patients with dementia due to Alzheimer disease

**Published:** 2016-06-30

**Authors:** Fabricio Ferreira de Oliveira, Juliana Marília Berretta, Elizabeth Suchi Chen, Marilia Cardoso Smith, Paulo Henrique Ferreira Bertolucci

**Affiliations:** 1 Department of Neurology and Neurosurgery, Escola Paulista de Medicina, Federal University of São Paulo (UNIFESP), São Paulo, SP, Brazil; 2 Department of Medicine, Escola Paulista de Medicina, Federal University of São Paulo (UNIFESP), São Paulo, SP, Brazil; 3 Department of Morphology and Genetics, Escola Paulista de Medicina, Federal University of São Paulo (UNIFESP), São Paulo, SP, Brazil

**Keywords:** Alzheimer disease, pharmacogenetics, urea, creatinine, Renin-Angiotensin System

## Abstract

**Background:**

Renal function declines according to age and vascular risk factors, whereas few data are available regarding genetically-mediated effects of anti-hypertensives over renal function.

**Objective:**

To estimate urea and creatinine variations in dementia due to Alzheimer disease (AD) by way of a pharmacogenetic analysis of the anti-hypertensive effects of angiotensin-converting enzyme inhibitors (ACEis).

**Methods:**

Consecutive outpatients older than 60 years-old with AD and no history of kidney transplant or dialytic therapy were recruited for prospective correlations regarding variations in fasting blood levels of urea and creatinine in one year, considering *ACE* genotypes of rs1800764 and rs4291 and their respective haplotypes, and treatment with ACEis along with blood pressure variations.

**Results:**

For 190 patients, 152 had arterial hypertension, and 122 used ACEis. Minor allele frequencies were 0.492 for rs1800764-C and 0.337 for rs4291-T, both in Hardy-Weinberg equilibrium. There were no overall significant yearly variations in levels of urea and creatinine, but their concurrent variations were positively correlated (*ρ* <0.0001). Each A allele of rs4291 led to an yearly urea increase of 3,074 mg/dL, and an yearly creatinine increase of 0.044 mg/dL, while the use of ACEis was protective regarding creatinine variations. The use of ACEis was also protective for carriers of rs1800764-CT/rs4291-AA, while carriers of rs1800764-CT/rs4291-AT had steeper reductions in creatinine levels, particularly when they were treated with ACEis.

**Conclusions:**

Effects of ACEis over creatinine variations are genetically mediated and independent of blood pressure variations in older people with AD.

## Abstract (es)

**Antecedentes:**

El deterioro de la función renal depende de la edad y los factores vasculares. La literatura sobre los efectos de fármacos antihipertensivos mediada genéticamente en la función renal es pobre.

**Objetivo:**

Estimar las variaciones de urea y creatinina a través del análisis farmacogenético de los efectos antihipertensivos de los inhibidores de la enzima convertidora de angiotensina (iECA) en pacientes con demencia debido a la enfermedad de Alzheimer.

**Métodos:**

Fueron reclutados pacientes consecutivos mayores de 60 años de edad con enfermedad de Alzheimer y sin antecedentes de trasplante renal o diálisis. Se determinaron correlaciones prospectivas durante un año entre los cambios en los niveles sanguíneos de urea y creatinina, considerando genotipos y haplotipos de *ACE* (rs1800764 y rs4291) y el tratamiento con iECA a y las variaciones en la presión arterial.

**Resultados:**

De 190 pacientes, 152 presentaron hipertensión, 122 usaron iECA. Las frecuencias de alelos polimórficos fueron de 0.492 para rs1800764-C y 0.337 para rs4291-T, los dos alelos en equilibrio de Hardy-Weinberg. No se determinaron fluctuaciones anuales significativas en los niveles de urea o creatinina, pero sus variaciones concomitantes se asociaron fuertemente (*ρ=* <0.0001). Cada alelo A de rs4291 condujo a aumentos anuales de 3,074 mg/dL en urea y 0.044 mg/dL en creatinina, mientras que el uso de iECA fue protector para las variaciones en la creatinina. El uso de iECA también fue protector para las personas con rs1800764-CT/rs4291-AA, mientras que los portadores de rs1800764-CT/rs4291-AT tuvieron reducciones de creatinina más altas, particularmente cuando se usó iECA.

**Conclusión:**

Los efectos de iECA en la variación de la creatinina son genéticamente mediadas e independiente de las variaciones en la presión arterial en pacientes de edad avanzada con la enfermedad de Alzheimer.

## Introduction

Renal function declines with age and according to the burden of vascular risk factors, while serum creatinine and proteinuria have been associated with late life incident all-cause dementia 1. The angiotensin-converting enzyme modulates the generation of angiotensin II, a vasoconstrictor that may lead to glomerulopathy by increased intraglomerular hydraulic pressure that can be improved by treatment with angiotensin-converting enzyme inhibitors 2. Both low and high glomerular filtration rates may be useful markers for mortality and cardiovascular events 3, while genetic influences might be important to mediate those risks.

Genetic effects account for most of the variance in serum levels of the angiotensin-converting enzyme 4. The two functional variants of the *ACE* gene with the most significant effects for higher activity of the angiotensin-converting enzyme are rs1800764 and rs4291 5. They also affect risk 6 and age at onset 7 of the amnestic phenotype of dementia due to Alzheimer disease, as well as cognitive decline 5: rs1800764 is located at ∼0.2 kb from the transcription start site in the promoter of *ACE* in 17q23, while rs4291 is at ∼3.8 kb from the same site 6. Both variants have local boosting effects upon serum angiotensin-converting enzyme levels, and have been strongly linked with risk of arterial hypertension 8,9, particularly for patients with coronary artery disease and cerebrovascular disease 10.

An earlier study had already demonstrated that the A1166C polymorphism of the *AT1R* gene may increase the anti-hypertensive effect of Benazepril 11. Nevertheless, pharmacogenetic effects of angiotensin-converting enzyme inhibitors over the age-related decline in renal function have never been studied before, even though they seem to boost genetically mediated neuroprotective effects in dementia due to Alzheimer disease 5 while also benefitting learning abilities in healthy older individuals 12. We aimed to estimate the variation in one year of levels of urea and creatinine in older people with dementia due to Alzheimer disease by way of a pharmacogenetic analysis of the effects of angiotensin-converting enzyme inhibitors, while taking into account possible impacts of systolic and diastolic blood pressure disparities over such variations.

## Materials and Methods

### Participants and clinical assessment

Consecutive outpatients with dementia due to Alzheimer disease according to National Institute on Aging - Alzheimer's Association criteria 13 were prospectively recruited from October 2010 to May 2014 at the Behavioural Neurology Section of our university hospital. Each patient had to be at least 60 years-old, and could not have history of kidney transplant or be receiving any form of dialytic therapy. All patients were followed for one year.

After diagnostic confirmation, all patients had at least three yearly consultations, and were assessed for age, gender, arterial hypertension, and use of angiotensin-converting enzyme inhibitors. For statistics, only the first and the last evaluations were taken into account, the last evaluation one year apart from the first one. Fasting urea and creatinine levels were measured from venous blood samples at the beginning and at the end of the follow-up after one year. Blood pressure was measured in every evaluation after the participant sat resting for 5 min in a warm and quiet room, while the diagnosis of arterial hypertension followed the JNC 7 report 14. All efforts were directed to control blood pressure for all patients rather than just lowering systolic and diastolic levels.

### Outcome measures

The primary outcome measure was the variation in one year of levels of urea and creatinine, taking the following independent variables into account: use of an angiotensin-converting enzyme inhibitor and *ACE* genotypes or haplotypes. In secondary analyses, the impacts of systolic and diastolic blood pressure variations in one year over variations in levels of urea and creatinine were assessed.

### Genotyping procedures

After blood samples were collected from all patients in tubes with ethylenediaminetetraacetic acid 0.1%, genomic DNA was extracted using a standard salting-out procedure. Genotyping was undertaken by way of Real-Time Polymerase Chain Reactions using TaqManENT#174; SNP Genotyping Assays on the Applied Biosystems 7500 Fast Real-Time PCR System (Applied BiosystemsENT#174;, CA, USA), following the standard protocols of the manufacturer. Presence of *ACE* genotypes of rs1800764 or rs4291, or their represented haplotypes, was correlated with anti-hypertensive treatment using angiotensin-converting enzyme inhibitors. All genotyping procedures were carried out only after clinical data were collected from all patients.

### Statistical analysis

One-way analysis of variance with post-hoc Tukey test was employed for variations of blood pressure, urea and creatinine in one year, taking initial and final values into account. The Hardy-Weinberg equilibrium for *ACE* genotypes was estimated by way of the *Chi*-square test. Multiple linear regressions were employed for comparisons within each patient group concerning variations of urea and creatinine in one year, with two degrees of freedom: *ACE* genotypes or haplotypes, and use or not of an angiotensin-converting enzyme inhibitor. Simple linear regressions were used for correlations between yearly variations in urea and creatinine, and yearly variations in systolic and diastolic blood pressure, so that genetically mediated effects of angiotensin-converting enzyme inhibitors over urea and creatinine variations could be evaluated independently of their blood pressure lowering properties. The threshold of significance was set at *p*= <0.05.

### Ethical considerations

This study is part of the research project 1067/10 (CAAE 0540.0.174.000-10) approved by the Ethics Committee of our university hospital in August 2010. Procedures of the study were in accordance with the Declaration of Helsinki. All invited patients and their legal representatives agreed to participate on the research and signed the Informed Consent Form before the evaluation.

## Results

A total of 217 patients were included in this study. During follow-up, 14 patients (6.5%) passed away, 11 patients (5.1%) abandoned the study, and 2 patients (0.9%) were excluded due to incomplete clinical data, resulting in a final sample of 190 patients.

Table 1 shows demographic and clinical results for all patients. Less than two thirds of them used angiotensin-converting enzyme inhibitors for blood pressure control. Systolic and diastolic blood pressure levels were significantly lowered after one year, while no significant overall variations were found for urea or creatinine levels in venous blood.

**Table 1.** Demographic aspects and variations of cardiovascular measures in one year

Assessed Factors, n= 190		n (%)	Mean±SD*	Range	p**
Gender	Female	126 (66.3)	-	-	-
	Male	64 (33.7)	-	-	-
Age at inclusion in the study (years-old)		-	78.06±6.1	60.0-94.5	-
Urea (mg/dL)	Initial values	-	40.27±15.9	11.00-118.0	0.777
	Final values	-	40.73±16.5	14.00-115.9	
	Yearly variation	-	0.46±15.0	-79.00 to +89.0	-
Creatinine (mg/dL)	Initial values	-	0.98±0.3	0.34-2.40	0.989
	Final values	-	0.98±0.3	0.32-2.10	
	Yearly variation	-	-0.01±0.2	-0.70 to +0.78	-
Arterial hypertension		152 (80.0)	-	-	-
Systolic blood pressure (mmHg)	Initial values	-	131.89±17.4	90-180	<0.01
	Final values	-	120.00±15.3	90-180	
	Yearly variation	-	-11.89±17.3	-50 to +30	-
Diastolic blood pressure (mmHg)	Initial values	-	78.53±10.1	60-110	<0.01
	Final values	-	73.62±9.5	50-100	
	Yearly variation	-	-4.91±10.4	-40 to +30	-
Anti-hypertensive treatment with an angiotensin-converting enzyme inhibitor (mg/day)	Captopril	111	74.44±29.7	37.5-150.0	-
	Perindopril	3	6.67±2.3	4.0-8.0	-
	Enalapril	8	37.50±7.1	20.0-40.0	-

*SD = standard deviation.

**One-way analysis of variance with post-hoc Tukey test.

Table 2 shows genotyping results for all patients. Minor allele frequencies were 0.492 for rs1800764 (C) and 0.337 for rs4291 (T), with both variants in Hardy-Weinberg equilibrium. Of nine possible *ACE* haplotypes, six were represented in the sample.

**Table 2.** Genetic proportions for ACE genotypes and respective haplotypes

Assessed Genotypes and Haplotypes, n=190		n (%)	p*
rs1800764 genotypes	CC	51 (26.9)	0.148
	CT	85 (44.7)	
	TT	54 (28.4)	
rs4291 genotypes	AA	89 (46.9)	0.077
	AT	74 (38.9)	
	TT	27 (14.2)	
ACE** haplotypes	rs1800764 CC / rs4291 AA	7 (3.7)	-
	rs1800764 CC / rs4291 AT	17 (9.0)	-
	rs1800764 CC / rs4291 TT	27 (14.2)	-
	rs1800764 CT / rs4291 AA	28 (14.7)	-
	rs1800764 CT / rs4291 AT	57 (30.0)	-
	rs1800764 CT / rs4291 TT	0 (0.0)	-
	rs1800764 TT / rs4291 AA	54 (28.4)	-
	rs1800764 TT / rs4291 AT	0 (0.0)	-
	rs1800764 TT / rs4291 TT	0 (0.0)	-

* Hardy-Weinberg equilibrium (Chi-square test)

** ACE= angiotensin-converting enzyme gene

Table 3 shows urea and creatinine variations in one year according to genotype and haplotype frequencies for *ACE* polymorphisms and the use or not of angiotensin-converting enzyme inhibitors. No significant impacts over urea or creatinine variations were found regarding any independent genotypes of rs1800764. The presence of each A allele of rs4291 led to an yearly urea increase of 3,074 mg/dL, and an yearly creatinine increase of 0.044 mg/dL, while the use of angiotensin-converting enzyme inhibitors was protective regarding creatinine variations. With regard to creatinine variations for all studied haplotypes, the use of angiotensin-converting enzyme inhibitors was protective for carriers of rs1800764 CT / rs4291 AA, while carriers of rs1800764 CT / rs4291 AT had steeper reductions in blood creatinine levels, particularly when they were treated with angiotensin-converting enzyme inhibitors.

**Table 3.** Effects of angiotensin-converting enzyme inhibitors over variations of urea and creatinine in one year according to ACE genotypes and respective haplotypes.

Genotypes and represented haplotypes		Urea variations (mean±SD* in mg/dL)			Creatinine variations (mean±SD* in mg/dL)		
		Users of ACEis**	Non-users of ACEis**	Total		Users of ACEis**	Non-users of ACEis**	Total
rs1800764 genotypes	CC	-1.32±11.5	-0.90±12.7	-1.17±11.8	0.02±0.2	-0.05±0.2	-0.01±0.2
	CT	2.01±17.0	-2.61±19.1	0.60±17.7	-0.02±0.2	0.00±0.2	-0.01±0.2
	TT	-2.10±11.9	6.66±13.1	1.79±13.1	-0.06±0.2	0.12±0.2	0.02±0.2
	β (*p*)	-0.448 ( 0.563)‡		1.477 ( 0.315)†	0.016 ( 0.210)‡			0.016 ( 0.410)†
rs4291 genotypes	AA	0.95±15.7	5.63±12.8	2.68±14.8	-0.01±0.2	0.12±0.2	0.04±0.2
	AT	0.06±13.8	-2.46±19.2	-0.89±15.9	-0.02±0.2	-0.04±0.2	-0.03±0.2
	TT	-2.17±13.1	-5.86±9.0	-3.13±12.1	-0.02±0.2	-0.09±0.2	-0.04±0.2
	β (p)	2.996 ( 0.127)‡		-3.074 ( 0.045)†	0.057 ( 0.029)‡		-0.044 ( 0.028)†
rs1800764 cc / rs4291 AA	Yes (n= 7)	-3.00±9.4	0.00±14.7	-1.71±10.9	0.07±0.1	0.00±0.2	0.04±0.1
	No (n= 183)	0.21±14.7	1.17±16.1	0.55±15.2	-0.02±0.2	0.03±0.2	0.00±0.2
	β (p)	1.219 ( 0.836)‡		-2.262 ( 0.697)†	0.029 ( 0.233)‡		0.045 ( 0.555)†
rs1800764 cc / rs4291 AT	Yes (n= 17)	1.33±8.8	3.10±14.8	2.16±11.7	0.07±0.1	-0.04±0.0	0.02±0.1
	No (n= 173)	0.00±14.9	0.85±16.2	0.30±15.3	-0.02±0.2	0.04±0.2	0.00±0.2
	β (p)	0.909 ( 0.817)‡		1.867 ( 0.626)†	0.029 ( 0.256)‡		0.023 ( 0.647)†
rs1800764 cc / rs4291 TT	Yes (n= 27)	-2.17±13.1	-5.86±9.0	-3.13±12.1	-0.02±0.2	-0.09±0.2	-0.04±0.2
	No (n= 163)	-0.55±14.8	1.92±16.4	1.06±15.4	-0.02±0.2	0.04±0.2	0.01±0.2
	β (p)	1.537 ( 0.386)‡		-4.185 ( 0.180)†	0.035 ( 0.170)‡		-0.045 ( 0.275)†
rs1800764CT / rs4291 AA	Yes (n= 28)	5.82±20.0	4.33±12.1	5.50±18.4	0.03±0.2	0.15±0.2	0.06±0.2
	No (n= 162)	-1.16±12.8	0.80±16.3	-0.41±14.2	-0.03±0.2	0.02±0.2	-0.01±0.2
	β (p)	0.571 ( 0.124)‡		5.906 ( 0.054)†	0.024 ( 0.045)‡		0.067 ( 0.096)†
rs1800764 CT / rs4291 AT	Yes (n= 57)	-0.25±14.8	-4.69±20.6	-1.81±17.0	-0.05±0.2	-0.04±0.2	-0.04±0.2
	No (n= 133)	0.25±14.5	3.53±13.1	1.44±14.0	0.00±0.2	0.06±0.2	0.02±0.2
	β (p)	2.067 ( 0.360)‡		-3.244 ( 0.173)†	0.049 ( 0.035)‡		-0.063 ( 0.043)†
rs1800764 TT / rs4291 AA	Yes (n= 54)	-2.10±11.9	6.66±13.1	1.79±13.1	-0.06±0.2	0.12±0.2	0.02±0.2
	No (n= 136)	0.82±15.3	-1.91±16.7	-0.06±15.7	-0.01±0.2	-0.02±0.2	-0.01±0.2
	β (p)	0.496 ( 0.699)‡		1.855 ( 0.444)†	0.021 ( 0.185)‡		0.033 ( 0.294)†

* SD = standard deviation

** ACEis = angiotensin-converting enzyme inhibitors.

‡ Coefficient for pharmacological treatment regarding multiple linear regressions with two degrees of freedom: genotype or haplotype variations and use or not of an angiotensin-converting enzyme inhibitor

† Coefficient for each T allele regarding simple linear regressions between genotypes or haplotypes and urea or creatinine variations

Overall, no correlations were found between urea variations and variations in systolic blood pressure (*p*= 0.212) or diastolic blood pressure (*p*= 0.098) in one year. Likewise, no correlations were found between creatinine variations and variations in systolic blood pressure (*p*= 0.767) or diastolic blood pressure (*p*= 0.713) in one year. Nevertheless, variations of urea and creatinine in one year were positively correlated with each other (*p=* <0.0001).

## Discussion

Our results showed that, despite the absence of significant overall variations for urea or creatinine, carriers of the A allele of rs4291 had higher age-related increases in urea and creatinine in one year, while the use of angiotensin-converting enzyme inhibitors was protective regarding creatinine variations. The use of angiotensin-converting enzyme inhibitors was also protective regarding creatinine variations for carriers of rs1800764 CT / rs4291 AA, but it was deleterious for carriers of rs1800764 CT / rs4291 AT. These results were independent of yearly variations in systolic or diastolic blood pressure, and it seems that rs4291 is more important for urea and creatinine variations than rs1800764.

The A allele of rs4291 has been associated with increased serum levels of the angiotensin-converting enzyme 9, while one study reported increased expression of *ACE* gene products with this allele 15. Associations with the A allele of rs4291 have also been found for lower fasting glucose, while angiotensin-converting enzyme inhibitors have been associated with risk reduction of incident diabetes *mellitus* 16. On the other hand, the C allele of rs1800764 has been associated with increased risk for diabetic nephropathy 2. These findings suggest independence between genetic pathways that mediate glucose homeostasis and renal function.

An earlier study found that people with dementia tend to experience greater late-life decreases in systolic and diastolic blood pressures 17. This could explain the significant disparities in blood pressure variations in our findings, though our close follow-up with aggressive anti-hypertensive therapy might also have affected these results. On the other hand, we found most genetic associations with creatinine rather than with urea levels. Since urea levels in blood may be affected by dietary protein and liver function, and not only by renal function, creatinine is considered a better indicator of glomerular filtration rates, thus explaining the outcomes we found.

Overall, 80% of our patients with dementia due to Alzheimer disease had arterial hypertension, confirming the burden of this risk factor over cardiovascular health in older people. Though we conveniently employed a cohort of patients who had dementia due to Alzheimer disease, the neurological diagnosis probably had little effect over the cardiovascular burden of our population. Nonetheless, earlier studies had confirmed the combined effect of cerebrovascular risk factors (arterial hypertension, diabetes *mellitus*, hypercholesterolemia, smoking and alcohol use) over earlier dementia onset^18^ and slower cognitive decline 19 for people with dementia due to Alzheimer disease, possibly related to atherosclerotic mechanisms throughout life and enhanced cerebral perfusion pressure in late life, respectively. Despite the genetically mediated neuroprotective effects of angiotensin-converting enzyme inhibitors in dementia due to Alzheimer disease 5, we have now shown that therapy of arterial hypertension with angiotensin-converting enzyme inhibitors may also protect the glomerular filtration of creatinine for carriers of specific genotypes, thus justifying the preferential use of these drugs as anti-hypertensive therapy for such patients.

Limitations of this study include the fact that it was conducted in a single centre, with a short follow-up and no randomization, and lacking measurements of serum levels of the angiotensin-converting enzyme or urinary creatinine clearance, as well as an evaluation of sarcopenia. Also, it is unknown whether the pharmacogenetic effects of angiotensin-converting enzyme inhibitors over urea and creatinine variations are either dose-dependent or more significant when starting treatment or at any time during anti-hypertensive therapy, due to the fact that many patients were already under treatment when they were included in the study. We tried to minimize these limitations by keeping observers blinded to genetic data during the evaluations. Nevertheless, to the best of our knowledge, this is the first study to evaluate the pharmacogenetic effects of angiotensin-converting enzyme inhibitors over age-related urea and creatinine variations.

## Conclusions

We conclude that the effects of anti-hypertensive therapy with angiotensin-converting enzyme inhibitors over creatinine variations are genetically mediated and independent of systolic or diastolic blood pressure variations. Future studies should assess pharmacogenetic effects of angiotensin-converting enzyme inhibitors regarding variations in urinary creatinine clearance, and consider serum levels of the angiotensin-converting enzyme as a variable.
